# Progress in Genito-Urinary Surgery

**Published:** 1901-01-12

**Authors:** 


### PROGRESS IN GENITO-URINARY SURGERY.

**Surgery of the Ureters.**—Malcolm L. Harris, of Chicago,[1] has contributed a paper on the best method of collecting urine from the ureters for diagnostic purposes. He remarks that all methods may be discarded except two, viz., those with the ureteral catheter and with the urine segregator. The segregator takes advantage of the separate points of issue of the ureters into the bladder, and mechanically prolongs them to the exterior of the body, segregating the urine into separate vials. The merits and demerits of the two methods are then considered. For cystoscopic catheterisation there must be a urethra of sufficient size to allow of the introduction of the instrument; and the bladder must be able to hold 120 to 150 cubic centimetres of fluid, and the fluid must remain transparent sufficiently long to permit the catheter to be introduced. Even if the above essential conditions are fulfilled there may still be intravesical obstacles to the introduction of the catheter, *e.g.*, the mouths of the ureters may not be found, or may be unapproachable, or be too small to admit the finest catheters. Extravesical conditions may distort the base of the bladder and prevent catheterisation. When the catheter has been introduced the urine may still fail to flow for various reasons, such as plugging of the eye of the catheter, or spasm of ureter with complete oliguria. The percentage of failures even in expert hands is large. Thus F. Tilden Brown is quoted as having reported failures in 15 per cent. of fifty-three cases tried. Again a certain amount of blood in the urine has to be disregarded, because the catheter produces it. Similarly, epithelial scales may be scraped from the ureters. Further, all the urine secreted from the kidney does not pass along the catheter, some flowing past it into the bladder, so that a fallacy is introduced in estimating the "functional capacity" of the kidney. Finally, there is a great danger in many cases of carrying infection from the bladder to the ureter or kidney, so that if cystitis is present, or tuberculosis of the bladder, catheterisation of the ureters is contra-indicated. The urine segregator is next considered. Again the bladder and urethra must be of a certain size, but the urine need not be clear as a view of the ureteral orifices is not required. The use of the segregator is interfered with by large fungus growths, by distortion of the base of the bladder as by unilateral enlargement of the prostate, by the trigone when it is elevated to form a septum, or by the middle lobe of the prostate projecting into the bladder. In post-prostatic pouch Harris thinks the segregator could be used. He then cites cases which illustrate the clinical results obtained with it. He has demonstrated that the septum of the segregator reliably separates the urine of one ureter from that of the other, if the correct rules are followed in using the instrument The instrument will not serve to differentiate renal from vesical disease if the urine collected becomes contaminated with pathological products formed within the bladder. The bladder and posterior urethra must be thoroughly irrigated and the prostate massaged at the same time By this means contamination of the urine as it passes from the ureters to the catheters can be sufficiently guarded against. The segregator may excite a little hæmorrhage from the bladder. Fresh solution of extract of supra-renal capsule will diminish or prevent the oozing of blood. An advantage of the segregator is that all of the urine which escapes from the ureters is collected in the vials, none being lost. There is no danger of infecting the kidney. By catheterisation of the ureters the nature and location of an obstruction in a ureter may be determined, the ends of a divided ureter may be found, fluid may be drained from the renal pelvis, or the renal pelvis irrigated, and the segregator does none of these things. But for many other purposes the latter instrument presents advantages.

J. Wesley Bovere[1] has published a paper on ureteral implantations. After accidental division the upper part has been implanted into the lower, or into the bladder, the urethra, the rectum, the colon, the cæcum, or the vagina, or even to the skin. These anastomoses are now being done for other conditions than complete section of the ureters, *e.g.* for ectopia vesicæ, for tumours of the bladder requiring its partial or complete removal, and for congenitally, abnormal ureteral endings. For severed ureter there are four principal methods of uniting the ends, viz., the transverse end to end, the oblique end to end, the end in end and the end in side. The writer then alludes to all the published cases of the modes of treatment. Of the operation of uretero-cystostomy Bovere found eighty cases recorded or quoted. The large number of cases of uretero-vaginal and other ureteral fistulæ account for the number of the cases. The mortality of rectal transplantation is very great, and 40 per cent. of the fatal cases are due to kidney infection. The other implantations of the ureters, viz., into the vagina, skin, and urethra are considered, and a complete bibliography is given. Dr. Howard Kelly[2] has also written a paper on uretero-ureterostomy and uretero-cystostomy. He has constructed a ureteral guide to overcome some of the difficulties of the operation. This is passed through a slit in the upper segment of the ureter two centimetres from the extremity, and the guide is passed through this, and through the severed ends, and loosely tied in the lower segment. With this guide in place the ureter can be rotated from side to side by moving its handle. When the suturing is completed the guide is removed.

**The Catheter Cystoscope.**—Schlagintwait[3] has devised a modification of the cystoscope to which the above name is applicable. The object of his device is to overcome the necessity for the removal and re-insertion of the cystoscope, and to render unnecessary the previous introduction of a catheter. His cystoscope is really an ordinary metal catheter, with the usual curve, carrying a lamp at its point, through which fluid can flow into and out of the bladder, and into which at will the telescope part of the ordinary cystoscope can be pushed down so that the prism lies in the eye of the catheter. As often as necessary the telescope part may be removed and the bladder washed out. A smaller field is visible with this instrument than with the ordinary cystoscope.

**Cystitis caused by the Bacillus Pyocyaneus.**—Thomas R. Browne of Baltimore[4] records an example of this. He points out that this bacillus was regarded as purely saprophytic until comparatively recently. But it is now known that the bacillus alone has caused a general septicæmia in a number of cases, and also wound suppuration, otitis media, puerperal infections, diphtheria-like infections of the larynx, endocarditis, hepatic abscess, pleuritis, impetigo, and ecthyma. Lartigau is quoted as having proved the bacillus to have been the cause of an epidemic dysentery. The case is the following:—Mrs. R. was operated upon by Dr. Kelly, an adherent Graafian follicle cyst of moderate size being removed. The bladder was subjected to traumatism during the proceedings. During the first few days the catheter was used but not after that. On the ninth day the temperature rose slightly and for nineteen days rose daily to 99° and 99·5°. Coincidently with the rise in temperature the patient complained of severe pain in the bladder, and pain and difficulty in voiding the urine, while the urine had a thick sediment and an unpleasant odour. Enormous numbers of pus cells, red-blood cells, myriads of mobile bacilli, and numerous epithelial cells were found. A specimen of urine was obtained with aseptic precautions, and agar plates prepared from it. Two or three hundred colonies of the bacillus pyocyaneus developed in twenty-four hours and the agar became blueish-green in colour. The urine became greenish in colour on standing from night to morning. The cystoscope was used, and showed general reddening of the vesical mucosa. Seventeen days later another culture was taken, and again a pure culture of the same bacillus obtained. Improvement was slow under treatment with borax and soda irrigations, so urotropine was given in five-grain doses three times a day, and within a few days of this the pus disappeared and cultures were negative. The pigment pyocyanin was isolated from the earlier cultures, and found to possess typical peculiarities. Three cases of cystitis observed by others are quoted in which this organism was found in the urine, but they were chronic cases, and it was impossible to say whether the infection was caused by this organism originally. But in the case given above the urine was normal a day or two before, and it is obvious that the bacillus pyocyaneus was the cause of the cystitis. The trauma rendered infection easier, and the probable mode of entrance of the organism was by the catheter.

**Tuberculous Disease of the Bladder.**—Dr. Saxtorph (Copenhagen) discussed this subject at the Thirteenth International Congress of Medicine[5] in Paris. He remarked that vesical tuberculosis almost always arose from an old pulmonary tuberculous focus. The infection occurred in two ways: (1) the bacilli were carried by the blood stream to the kidney, whence they infected the bladder (descending infection); and (2) the bacilli were carried in the blood stream to the prostate, vesiculæ seminales, testicles, or epididymes, whence they infected the vesical wall by contiguity (ascending infection). Vesical tuberculosis being always of a secondary nature, there was no radical treatment except removal of the source of infection by nephrectomy and more or less resection of the ureter, or by destruction of the tuberculous foci in the genital organs. If this treatment could not be carried out radical treatment of vesical tuberculosis was useless. If the infective centre was removed the bladder disease might undergo spontaneous cure or might be treated surgically, viz. by supra-pubic opening and removal of the diseased mucous membrane with the scissors, cautery or curette. The patient should be in Trendelenburg's position. Similar treatment might be adopted to relieve pain where radical cure was not possible, and in the female the curetting might be done through the urethra.

Dr. Hogge (Liège) followed, and discussed the surgical treatment of tuberculosis of the prostate, testicles, and vesiculæ seminales. For the testicular disease total castration appeared to be only exceptionally required, resections being usually better. If the disease of the prostate, vas deferens, vesiculæ, epididymis, or tes ticle, led to abscess or sinus formation, operation should be the rule. Results seemed to show that surgeons would do well to adopt free extirpation.

**Exstrophy of the Bladder.**—Professor John Berg (Stockholm)[6] contributes a paper on this subject based upon an experience of the treatment of 18 cases. All the attempts hitherto made to remedy this condition are different modifications of the three principal methods indicated by Dubois, Dupuytreu, Roux, and John Simon. These were (1) The plan of directly joining the sound edges of the bladder and of the abdominal wall; (2) The method of completing the bladder by means of transplantation; and (3) The radical method of transplanting the ureters and excising the bladder. In eight cases Berg divided the iliac bones in a vertical direction, thus modifying the synchondroseotomy of Trendelenburg, in order to diminish the diastasis of the symphysis pubis. To keep the bones in the corrected position, he used ivory wedges placed between the thickest parts of the osseous surfaces, and found much benefit from them, and saved the patients the discomfort of compressing bandages. The diastasis was not completely done away with, an interval of 1 cm. to 2 cm. generally remained. In one of his latter cases the three operations were done at intervals of ten days. In some cases he found it best not to make any attempt to directly join the parts but to use a method of transplantation he describes. The flap which he has used since 1890 is a little larger than the opening in the abdominal wall, and is dissected up from the groin with its shaft directed down and inwards close to the borders of the ectopic bladder. The flap is turned down towards the thigh and fastened there with a few stitches with the raw surface forwards. This surface is covered with grafts after the method of Thiersch, and left for eight to fourteen days, and then, after its edges and those of the bladder have been roughened, it is surtured n its place, so that its deep surface comes into continuity with the bladder mucous membrane, and its front with the abdominal skin. Berg has used a similar method in remedying great defects of the mouth, nose, eyelids, and uretha. It gives a good substitute for mucous membrane, and does not favour incrustations. As to results, they were the best where direct union could be obtained after approximation of pubic bones, in which case a bladder lined entirely with mucous membrane was obtained. Such a bladder may hold up to 250 grams of urine, and may possess good powers of possible retention. Several patients could during work hold urine three to six hours by means of a bandage slightly pressing against the radix penis; others still preferred to use a urinal. Berg considers that Simon's operation will be found to supersede other methods in a number of cases.

[1] An. of Surg., Aug. 1900. [2] Journ. of Amer. Med. Assoc., Oct. 1900. (Abstracted in Lancet, Nov. 1900.) [3] Vide Scot. Med. and Surg. Journ., vol. vii. No. 3. [4] The Therapist, Sept. 1900. [5] Vide Lancet, Aug. 1900. [6] Brit Med. Jour., Oct. 1900.

